# New Monoclonal Antibodies to Defined Cell Surface Proteins on Human Pluripotent Stem Cells

**DOI:** 10.1002/stem.2558

**Published:** 2017-01-19

**Authors:** Carmel M. O'Brien, Hun S. Chy, Qi Zhou, Shiri Blumenfeld, Jack W. Lambshead, Xiaodong Liu, Joshua Kie, Bianca D. Capaldo, Tung‐Liang Chung, Timothy E. Adams, Tram Phan, John D. Bentley, William J. McKinstry, Karen Oliva, Paul J. McMurrick, Yu‐Chieh Wang, Fernando J. Rossello, Geoffrey J. Lindeman, Di Chen, Thierry Jarde, Amander T. Clark, Helen E. Abud, Jane E. Visvader, Christian M. Nefzger, Jose M. Polo, Jeanne F. Loring, Andrew L. Laslett

**Affiliations:** ^1^ Clayton and ParkvilleCSIRO ManufacturingVictoriaAustralia; ^2^ Australian Regenerative Medicine Institute, Monash UniversityClaytonVictoriaAustralia; ^3^ Department of Anatomy and Developmental BiologyMonash UniversityClaytonVictoriaAustralia; ^4^ The Walter and Eliza Hall Institute (WEHI)ParkvilleVictoriaAustralia; ^5^ Department of Medical Biology; ^6^ Department of MedicineThe University of MelbourneParkvilleVictoriaAustralia; ^7^ Department of SurgeryCabrini Monash UniversityMalvernVictoriaAustralia; ^8^ Department of Chemical Physiology; ^9^ Center for Regenerative Medicine, The Scripps Research InstituteLa JollaCaliforniaUSA; ^10^ Department of Medical OncologyThe Royal Melbourne HospitalParkvilleVictoriaAustralia; ^11^ Department of MolecularCell and Developmental Biology, University of CaliforniaLos AngelesCaliforniaUSA; ^12^ Cancer Program, Monash Biomedicine Discovery Institute; ^13^ Centre for Cancer Research, Hudson Institute of Medical ResearchClaytonVictoriaAustralia

**Keywords:** Pluripotency, Human embryonic stem cells, Human iPS cells, Naive, Breast, Colorectal, Cell surface markers, Monoclonal antibodies, Cancer

## Abstract

The study and application of human pluripotent stem cells (hPSCs) will be enhanced by the availability of well‐characterized monoclonal antibodies (mAbs) detecting cell‐surface epitopes. Here, we report generation of seven new mAbs that detect cell surface proteins present on live and fixed human ES cells (hESCs) and human iPS cells (hiPSCs), confirming our previous prediction that these proteins were present on the cell surface of hPSCs. The mAbs all show a high correlation with POU5F1 (OCT4) expression and other hPSC surface markers (TRA‐160 and SSEA‐4) in hPSC cultures and detect rare OCT4 positive cells in differentiated cell cultures. These mAbs are immunoreactive to cell surface protein epitopes on both primed and naive state hPSCs, providing useful research tools to investigate the cellular mechanisms underlying human pluripotency and states of cellular reprogramming. In addition, we report that subsets of the seven new mAbs are also immunoreactive to human bone marrow‐derived mesenchymal stem cells (MSCs), normal human breast subsets and both normal and tumorigenic colorectal cell populations. The mAbs reported here should accelerate the investigation of the nature of pluripotency, and enable development of robust cell separation and tracing technologies to enrich or deplete for hPSCs and other human stem and somatic cell types. Stem Cells *2017;35:626–640*

**Significance Statement**

This manuscript describes the production and characterization of seven new monoclonal antibodies. These antibodies are significant because they enable new explorations of human pluripotent stem cells. The antibodies should be useful for gaining a better understanding of human pluripotency, enriching for human pluripotent stem cells, removing pluripotent stem cells, and also for performing similar functions with subsets of human breast and colon tissue.

## Introduction

Human ESCs 1 and iPSCs 2, 3 have revolutionized the possibilities for cell‐based regenerative therapies, but there are few in vitro diagnostic approaches that facilitate quality control of live human pluripotent stem cells (hPSCs) and their derivatives. Refinement of such approaches is an essential requirement for the safe and effective translation to diagnostic and therapeutic applications of hPSC‐derived cell types 4, 5. Strategies ranging from cytotoxic and proapoptotic chemicals 6, 7, 8, 9, 10 to cell‐surface biomarkers are being used to remove undifferentiated hPSCs from lineage‐differentiated cells 11, 12. Monoclonal antibodies (mAbs) are especially useful because of their sensitivity and specificity. However, many of the conventionally used cell surface markers for hPSCs are not reactive with proteins, but instead recognize complex carbohydrate or lipid moieties for which there are no identified corresponding genes 13. Some of the markers used for characterization of hPSC lines are also immunoreactive in mature cell types, so are useful only within a limited time frame of hPSC culture 11. In recent years, there have been additional markers developed that are reported to be highly specific for detecting hPSC surface proteins or glycans 14, 15, 16, 17, 18, 19 but few of these are directed against known proteins. Having a variety of antibodies would be useful for purifying conventionally cultured hPSCs that are lineage primed, akin to mouse epiblast‐derived cells 20, 21, display heterogeneity and have been demonstrated to contain cell populations that express varying levels of pluripotency‐associated markers 19, 22, 23, 24, 25, 26, 27, 28. Moreover, availability of new pluripotency cell‐surface markers will aid investigation of the distinctive naive state that has been recently described for human PSC cultures 29, a state more similar to the ground state of inner cell mass (ICM)‐derived mouse ESCs 30. Therefore, strategies using new cell‐surface markers to investigate human pluripotency, to help resolve the heterogeneity that occurs with in vitro hPSC culture, as well as to stringently detect and eliminate all undifferentiated cells from enriched differentiated populations would be extremely valuable to the field.

## Materials and Methods

### Informed Consent

All work using hPSCs was carried out in accordance with approvals from Monash University and the CSIRO Human Research Ethics Offices or by the full UCLA Institutional Review Board and the UCLA Embryonic Stem Cell Research Oversight Committee. Human breast tissue (pathologically normal) from reduction mammoplasty surgeries was donated by consenting individuals through the Victorian Cancer Biobank under the approval of the WEHI and Melbourne Health Human Research Ethics Committees. Human normal colon and colorectal cancer tissues were resected from consenting individuals through the Cabrini Hospital under the approval of the Cabrini Human Research Ethics Committee.

### Animal Care

Protocols and use of animals in this project were undertaken with approval of the Monash University Animal Welfare Committee following the Australian Code for the Care and Use of Animals for Scientific Purposes (8th Edition 2013) and the Victorian Prevention of Cruelty to Animals Act and Regulations legislation.

### HPSC Culture and In Vitro Differentiation

Undifferentiated, karyotypically normal MEL1 31, WA09 [1] (H9, WiCell Research Institute, Madison, WI, http://www.wicell.org) human ES cell (hESC) lines and hiPS‐PDL‐D1C6 32, hiPS‐NHF1.3 33 human iPS cells (hiPSC) lines were routinely maintained on a monolayer (1.2 × 10^4^ cells/cm^2^) of mitotically inactivated mouse embryonic fibroblasts (MEFs) in hPSC medium comprised of Dulbecco's modified Eagle medium:nutrient mixture F‐12 (DMEM/F12, 1:1), 20% v/v knockout serum replacement (KOSR), 2 mM Glutamax, 1% v/v modified Eagle's medium (MEM) nonessential amino acids (NEAA), (all from Life Technologies, Carlsbad, CA, http://www.lifetechnologies.com), supplemented with 10 ng/ml human basic fibroblast growth factor (bFGF, Merck Millipore, Billerica, MA, http://www.merckmillipore.com). For some experiments hPSC lines were grown in Essential E8 Medium on a Geltrex Matrix (all from Life Technologies) 34, 35.

Cells were cultured (37°C/5% CO_2_ in air) to 70%‐80% confluence with daily hPSC media changes then passaged using 300 U/ml Collagenase I (Worthington Biomedical Corp., Lakewood, NJ, http://worthington-biochem.com) in DMEM/F12, (0.5 mM EDTA for Essential E8 cultures), washed and replated at dilutions of 1:4‐1:10. Nondirected in vitro differentiation of hPSC cultures was performed using an embryoid body (EB) method as previously described in detail 36. EBs were collected following 7, 14, and 28 days of differentiation for flow cytometric analyses as described below. A routine hPSC maintenance culture provided a day 0 control for each differentiation time course analysis.

To investigate mAb detection of naive state pluripotent cells, hiPSCs were generated from two different human adult dermal fibroblast (HDF) cell lines (Life Technologies, C‐013‐5C: 1528526, 569390), using the Cytotune II kit (Life Technologies) according to the manufacturer's instructions, and cultured initially in the standard hPSC culture conditions described above and then in three different chemically defined culture media reported to reset cells to a naive phenotype. The media used were Naive Human Stem Cell Medium (NHSM) 37, 5i/hLIF medium supplemented with FGF and Activin A (5i/L/FA) 38 and RSeT medium (StemCell Technologies, Vancouver, Canada, http://www.stemcell.com), all of which have been reported to successfully culture naive state hPSCs 37, 38. Additionally, mAb detection using a human embryonic stem cell line UCLA20n 39 derived using 5i/L/FA medium was investigated.

To harvest undifferentiated (primed and naive) hPSC cultures and differentiated EBs for flow cytometric analyses and fluorescence activated cell sorting (FACS), cultures were washed twice with Dulbecco's phosphate buffered saline (PBS) without calcium and magnesium (CMF‐PBS) and dissociated to single cell suspensions using TrypLE Express (all from Life Technologies) in 5‐8 minute (hPSCs) or 15‐30 minute (EBs) incubations (37°C/5% CO_2_ in air) with gentle pipetting. Single cell harvests were washed twice in DMEM/F12, resuspended in FACS buffer (as below) and kept on ice for immunolabeling.

### Somatic Human Cell Preparations

Human bone marrow‐derived MSCs (hBM‐MSCs) were sourced and propagated from cryopreserved stocks (Lonza, PT‐2501, Basel, Switzerland, http://www.lonza.com) cultured on tissue culture flasks coated with Geltrex Matrix (Life Technologies) according to manufacturers' instructions in MSC Growth Medium comprised of low glucose DMEM, 2 mM L‐Glutamine, 1 mM sodium pyruvate, 1% v/v antibiotic/antimycotic comprising 100 U/ml penicillin, 100 µg/ml streptomycin, 25 µg/ml amphotericin B (all from Life Technologies), supplemented with 20% v/v fetal bovine serum (FBS), (Sigma‐Aldrich, St. Louis, MO, http://www.sigmaaldrich.com). A complete media change was performed every 3‐4 days and the cells were passaged at ∼90% confluence. Human BM‐MSCs were were maintained at 37°C in a humidified atmosphere containing 5% CO_2_. Human mammary cell preparations were prepared as previously described 40 and were cryopreserved until required for immunostaining and flow cytometric analyses. For intestinal cell preparations, normal and cancerous human colorectal tissues were incubated, respectively, with a 3 mM EDTA‐0.25 mM DTT solution at 4°C for 45 minutes or a 0.125 mg/ml dispase‐1 mg/ml collagenase (Sigma‐Aldrich) solution at 37°C for 30 minutes. Following subsequent washes with CMF‐PBS and centrifugation, both normal and cancer cells were dissociated in TrypLE Express (Life Technologies) supplemented with 10 μM Rock inhibitor (Y‐27632, Abcam, Cambridge, UK, http://www.abcam.com) and DNAse 1 (Sigma‐Aldrich) for 2 minutes at 37°C.

### Antigen Generation

From our previously published list of genes predicted to express cell‐surface proteins on hPSCs 25, 40 candidate immunogens were selected for which antibodies were not commercially available at the time, or for which available antibodies could not detect the native protein for both live and fixed hPSCs by FACS and immunocytochemical (ICC) analyses (data not shown). The domain structure for selected hPSC membrane protein candidates was analyzed using the UniProt KnowledgeBase database (http://www.uniprot.org/) and antigens generated for the largest extracellular domain. Briefly, peptide synthesis (Auspep, http://www.auspep.com.au) was used to generate immunogens for small transmembrane domain proteins (<50 amino acids), building fragments of 19‐30 amino acids with no internal cysteine and predicted hydrophilicity, antigenicity, and surface probability. For medium (50‐200 amino acids) and large (>200 amino acids) domain proteins, corresponding coding regions, incorporating a C‐terminal Flag epitope tag, were synthesized by PCR, and cloned into mammalian expression vectors for transfection and transient expression in suspension‐adapted 293Freestyle cells (Life Technologies). Recombinant antigens were purified from scaled cultures by immunoaffinity and size exclusion chromatography. Gene identifier and amino acid sequence of targets for the seven mAbs described below are given in Supporting Information Table S1.

### Hybridoma Derivation and Culture

Hybridomas specific to hPSC antigens were generated at the Monash Antibody Technology Facility (MATF, Monash University, Melbourne, Australia, https://platforms.monash.edu/matf/). Briefly, CD1 mice were injected intraperitoneally with peptide or recombinant protein antigen corresponding to the selected target proteins. Following an ELISA serum titre confirmation, mice received a prefusion boost immunization using irradiated MEL1 hES cells, prior to isolation of B cells from the spleen and fusion to SP2/0 Ag‐14 mouse myeloma cells. Hybridomas were subjected to limited dilution in 96‐well plates (1,920 wells per fusion) and propagated for 13 days in Hybridoma medium (HM) comprised of high glucose DMEM, 2 mM Glutamax, 1% v/v penicillin‐streptomycin (all from Life Technologies), supplemented with 20% v/v FBS (HyClone, GE Healthcare Life Sciences, Chicago, IL, http://www.gelifesciences.com) and 1% v/v HybER murine IL‐6 (SSI Diagnostica, Hillerød, Denmark, http://www.ssi.dk/ssidiagnostica). Hybridoma supernatants were collected and initially screened by direct solid‐state antigen microarray assay (ArrayJet, Tecan, Männedorf, Switzerland, http://www.tecan.com) to identify hybridomas generating IgG antibodies binding each immunization protein. Array‐positive supernatants (up to 300 per target antigen) were immunolabeled and screened by flow cytometry using a LSRII flow cytometry analyzer equipped with a high throughput 96‐well plate module option (BD Biosciences, San Jose, CA, http://www.bdbiosciences.com). Hybridomas corresponding to live hPSC detection were expanded in HM medium/20% v/v FBS/1% v/v HybER and subclones raised from single cells robotically sorted for individual 24‐well plate culture (Tecan). Supernatants from clonal cultures were again immunolabeled and screened to confirm detection of live hPSCs by flow cytometry (see Supporting Information Fig. S1A). Parental and subcloned hybridoma cultures were expanded in HM medium/20% v/v FBS, passaging at subconfluence each 2‐4 days and eliminating HybER IL‐6 in a stepwise manner. Cells were cryopreserved in HM/20% v/v FBS with 1% v/v DMSO (Sigma‐Aldrich) and stored in vapor phase nitrogen.

### Purification of mAbs

To purify and concentrate antibody produced from subcloned hybridomas, cell cultures were expanded to confluence in 175 cm^2^ tissue culture flasks (Greiner Bio‐One GmbH, Frickenhausen, Germany, https://www.gbo.com) in HM medium without the addition of penicillin‐streptomycin and with a stepwise reduction of FBS from 20% v/v to 2.5%‐10% v/v, switching to an ultra‐low IgG FBS (Life Technologies) prior to the exhaustion of cultures. Supernatants were separated from cells by centrifugation, passed through a 45 µm filter (Sartorius Stedim Biotech, Goettingen, Germany, https://www.sartorius.com), and aliquots taken for isotyping (IsoStrip, Roche, Basel, Switzerland, http://www.roche,com) and repeat confirmation of live hPSC detection by FACS (see Supporting Information Fig. S1A). mAbs were purified from these culture supernatants. Briefly, IgG fractions were isolated by affinity chromatography using mAb Select Sure recombinant protein A and protein G sepharose HP columns (GE Lifesciences). Purified mAbs were concentrated using an Ultra‐15 centrifugal concentrator (Merck Millipore), then further purified by size exclusion chromatography on a Superdex 200 pg 16/60 column (GE Lifesciences) and concentrated as above, in CMF‐PBS containing 0.02% v/v azide. Purified mAb proteins were analyzed by SDS‐PAGE (see Supporting Information Fig. S1B) under both nonreduced and reducing conditions on 4%‐12% BisTris NuPAGE gels (Life Technologies).

### Antibodies

All mAbs generated as well as commercially available primary, conjugated and secondary antibodies used in this study are listed in Supporting Information Table S2.

### Immunostaining (Live and Fixed Cell) and Flow Cytometry

Undifferentiated hPSC, differentiated EB, human mammary and hBM‐MSC cell preparations were resuspended in cold FACS buffer comprised of either DMEM/F12 or CMF‐PBS supplemented with 10% v/v FBS (Sigma‐Aldrich) and for intestinal cell preparations, CMF‐PBS supplemented with 2 mM EDTA, 2% v/v FBS, and 10 μM Rock inhibitor (Y‐27632). Mammary cell suspensions were blocked with 10% v/v FBS, 0.1 mg/ml DNAseI, and anti‐CD16/CD32 Fcg III/II receptor antibody for 10 minutes at 4°C prior to staining. Cell suspensions were aliquoted for fluorescent labeling reactions including unstained cells, isotype and single color fluorophore controls, keeping all tubes on ice during hybridoma supernatant, antibody and lectin labeling procedures, and prior to flow cytometry and FACS analyses. Extracellular, intracellular, single, and multicolour immunolabeling reactions and FACS analyses were all performed as previously described 11, 40, 41, 42, 43. Briefly, cells were labeled for 30 minutes with hybridoma supernatants or purified mAbs raised to GPR64, CDCP1, F11R, DSG2, CDH3, NLGN4X, PCDH1 both singly and in combination with other cell‐surface markers CD9, SSEA‐3, SSEA‐4, TRA‐1‐60, GCTM‐2, and biotinylated Ulex Europaeus Agglutinin I (UEA‐I) lectin (see Supporting Information Table S2). Cells were next washed in FACS buffer and incubated for 30 minutes with conjugated fluorophore isotype matched secondary antibodies or streptavidin, again washed and resuspended in FACS buffer supplemented with 0.1% v/v propidium iodide (Sigma‐Aldrich) for the exclusion of nonviable cells at analysis. HPSCs were also immunostained for murine CD90.2, human TRA‐1‐85, EpCAM, or OCT4 to exclude MEF cells from FACS analyses except where MEFs expressing green fluorescent protein were used, and hBM‐MSCs were immunostained for human CD90 for phenotype validation. Note, for the first step biotinylated UEA‐I labeling, the FACS buffer was replaced with a 5% v/v ultrapurified BSA in Hank's Buffered Saline Solution (HBSS), (Life Technologies), for all wash steps and for diluting the streptavidin fluorophore to avoid potential reactivity between UEA‐1 and serum glycoproteins, or streptavidin with biotin. For OCT4 multicolor analyses, cells were sequentially live cell immunostained for cell‐surface markers followed by fixation, permeabilization and intracellular immunostaining with anti‐human OCT4, as previously described in detail 11, 42.

Immunostained mammary cells were further washed, blocked in 5% v/v mouse serum, 5% v/v rat serum (Sigma‐Aldrich), 0.1 mg/ml DNAseI, 1 mg/ml rat immunoglobulin, and anti‐CD16/CD32 Fcg III/II receptor antibody for 10 minutes then immunostained with conjugated primary antibodies EpCAM‐Pacific Blue (Clone VU1D9), CD49f‐PECy7 (Clone GoH3), CD45‐PE (Clone H130), CD31‐PE (Clone WM59), and CD235a (Clone GA‐R2) prior to resuspension in propidium iodide as above. Mammary cell subsets were delineated by FACS analysis, gating for a viable CD31^−^CD45^−^CD235a^−^ population to exclude hematopoietic and endothelial cells, then EpCAM and CD49f to yield luminal progenitor (CD49f^+^ EpCAM^+^), mature luminal (CD49f^−^ EpCAM^+^), MaSC and basal (CD49f^+^ EpCAM^−^), and fibroblast‐enriched stromal (CD49f^−^EpCAM^−^) cells. Intestinal cells were further immunostained with conjugated primary antibodies EpCAM‐BV421 (Clone EBA‐1), CD31‐BV510 (Clone WM59), and CD45‐BV510 (Clone HI30). For FACS analysis of gut samples we gated in on intestinal epithelial cells by selecting EpCAM^+^ cells while excluding contaminating endothelial (CD31^+^) and hematopoietic (CD45^+^) cells.

All fluorescently labeled cell suspensions were filtered through a 40‐µm filter mesh (BD Biosciences) and resuspended in relevant FACS buffer prior to performing multiple color analyses on a LSRII flow cytometry analyzer (BD Biosciences). Spectral compensation for auto and nonspecific fluorescence to determine fluorophore positive and negative cell populations was performed as previously described 11. FACS fractionation and replating of viable cells to colony‐forming assays (see below) was performed using Influx and FACSFortessa instruments (BD Biosciences). For investigating mAb detection of cells in the GCTM‐2/CD9 coexpression gradient found in hPSC cultures 23, hPSCs triple‐labeled for mAb/GCTM‐2/CD9 detection were analyzed using a FACS Diva Instrument (BD Biosciences) calibrated and gates set for GCTM‐2/CD9 negative and high coexpression populations as previously described 23, 41. Flow cytometric data was generated with instrument software (BD Biosciences) and analyzed using FlowJo software (Tree Star Inc.).

### ICC Staining and Imaging

hPSC cultures harvested using Collagenase I (colony clump) or Accutase (Life Technologies), (single cell) dissociation were cultured in sterile Multitest 12‐well (8 mm diameter) glass slide chambers (MP Biomedicals, Santa Ana, CA, http://www.mpbio.com) preseeded with MEF feeder cells and maintained in hPSC medium as described earlier. At subconfluence cultures were rinsed with CMF‐PBS, fixed with ice‐cold absolute ethanol for 5 minutes and air‐dried at room temperature, prior to storing at −20°C or directly proceeding to immunolabeling reactions. Fixed hPSCs were incubated for 30 minutes at room temperature in blocking buffer comprising CMF‐PBS supplemented with 10% v/v goat serum (Life Technologies). Cells were then incubated with the new purified mAbs against GPR64, CDCP1, F11R, DSG2, CDH3, NLGN4X, PCDH1 as well as SSEA‐3, TRA‐1‐60, GCTM‐2, and CD9 antibodies diluted in blocking buffer for 60 minutes at room temperature, washed twice in CMF‐PBS, then incubated with secondary Alexa Fluor conjugated antibody(s) diluted in blocking buffer, for 60 minutes at room temperature. Immunostained cells were washed twice in CMF‐PBS, nuclear counterstained for 5 minutes in 4′,6‐diamidino‐2phenylindole (DAPI), (Sigma‐Aldrich, 10 ng/ml in CMF‐PBS) and mounted in Vectashield (Vector Laboratories, Burlingame, CA http://www.vectorlabs.com). For the intracellular OCT4 immunostaining, cells were first stained with extracellular antibodies and then sequentially with the mouse anti‐human OCT4 antibody (Merck Millipore) diluted in blocking buffer, (see Supporting Information Table S2 for details of primary and secondary antibodies used). Fluorescence was observed using an Olympus BX51 inverted microscope and images captured using a Nuance multispectral imaging system 3.0.2 (Perkin Elmer, Waltham, MA http://www.perkinelmer.com). HPSC bright field colony images were taken using a Motic AE2000 light microscope and Motic Images Plus 2.0 software (Motic, Hong Kong, http://www.motic.com).

### Colony‐Forming Assays

HPSC colony‐forming assays were performed in 12‐well (3.8 cm^2^/well) tissue culture plates (BD Biosciences) as previously described 41 with the following modifications. HPSCs were immunolabeled with antibodies and lectin then fractionated by FACS, replating to culture cells from the top 25% of mAb‐fluorochrome positive events gated against isotype and unstained hPSC controls. Cells were plated per triplicate at a density of 5,000 cells per well and cultured in hPSC medium on supporting MEFs as described above. Colonies formed were counted manually on day 5 on a Nikon Eclipse T*i* microscope and cultures harvested enzymatically on day 7 and prepared for intracellular OCT4 immunolabeling and flow cytometry analyses as described above.

### RNA Sequencing

RNA was extracted using the RNeasy Micro Kit (Qiagen, Hilden, Germany, https://www.qiagen.com), according to the manufacturer's instructions, from 2‐3 × 10^4^ FACS‐purified hPSCs per sample. For the generation of sequencing libraries, 25 ng of RNA (RIN value >9) was subjected to SPIA amplification (NuGen). Two biological replicates per culture condition were sequenced using the HiSeq 2000 sequencing platform (Illumina, San Diego, CA, http://www.illumina.com). The targeted number of sequencing reads per sample was 30 million (50 bp single reads). RNA‐seq samples were deposited at the NIH Short Reads Archive (www.ncbi.nlm.nih.gov/sra), accession numbers SRP093689, SRP094406, and SRP094408.

### Bioinformatic Analysis

Sample sequencing reads were aligned to the human genome (complete hg19 [UCSC version, July 2007]) using Tophat2 (v 2.0.13, default parameters 44). Transcript quantification was performed using HTSeq (v 0.6.1, default parameters 45). Differential gene expression analysis was performed using limma 46 and edgeR 47. In summary, library size was normalized using voom 48, linear models were fit to transcripts and differential gene expression assessed using eBayes moderated *t* statistic. Significantly differentially expressed genes were selected on the basis of an absolute Log_2_ expression value of 1 and *p* < .05, adjusted for multiple testing to control false discovery rate using Benjamini and Hochberg's method 49. Normalized gene expression array values from naive and primed cells of Theunissen's et al. (2014) study were extracted from Supporting Information Table S1 of the published report 38. To compare array expression values versus RNA‐seq counts, platform‐specific effects were removed using limma's removeBatcheffect function on logarithmic base 2 transformed values.

### ELISA

Purified mAb detection for each corresponding immunogen to which hybridomas were initially raised was confirmed by ELISA, except for anti‐hGPR64 which was raised to a peptide immunogen. A 96‐well microplate (R&D Systems, Minneapolis, MN, http://www.rndsystems.com) was prepared with 100 µl of 1 mg/ml purified antigen protein per well and incubated overnight at 4°C. Anti‐hCDCP1 (20 µg/ml), anti‐hF11R (4 µg/ml), anti‐hDSG2 (3 µg/ml), anti‐hCDH3 (20 µg/ml), anti‐hNLGN4X (20 µg/ml), anti‐hPCDH1 (20 µg/ml) were added to corresponding antigen coated wells (100 µl/well), and the antigen‐mAb cross‐linking detected using secondary antibody (100 µl/well), Alexa Fluor488 conjugated goat anti‐mouse IgG (Life Technologies) at 1:500 dilution. The fluorescent measurement was monitored using the Enspire 2300 Multilabel Reader (Perkin Elmer) at 488 nm and 519 nm for excitation and emission, respectively.

### Data Analysis

All experimental assays (except where noted) were performed in triplicate at a minimum on biologically discrete cell samples. All data with error bars represent SEM, unless otherwise stated.

## Results and Discussion

### Target Selection and Generation of mAbs

**Figure 1:**
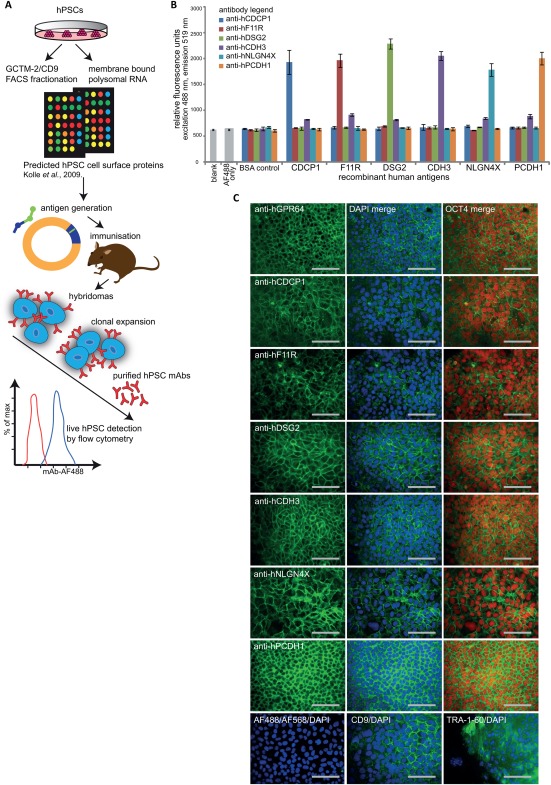
Monoclonal antibodies (mAbs) raised to candidate cell‐surface antigens detect epitopes on human pluripotent stem cells (hPSCs). **(A)**: A schematic overview for the approach used to generate new mAbs capable of recognising hPSC extracellular proteins. Target proteins were identified from fluorescence activated cell sorting (FACS)‐based CD9/GCTM‐2 immunotranscriptional profiling of hPSC cultures bioinformatically combined with membrane polysome translation state array analyses 25, and antigens generated by peptide synthesis or cloning into mammalian expression vectors. Hybridomas raised and initially validated by solid‐state antigen array were screened by high throughput flow cytometry for detection of live hPSCs, prior to cloning, expansion, mAb purification and final validation by detection of live hPSCs by flow cytometry. **(B)**: ELISA fluorescence measurement at 488 nm excitation and emission 519 nm validated specific detection of recombinant antigen immunogens corresponding to purified anti‐hCDCP1 (blue bar), anti‐hF11R (red bar), anti‐hDSG2 (green bar), anti‐hCDH3 (purple bar), anti‐hNLGN4X (light blue bar), anti‐hPCDH1 (orange bar) antibodies compared with control wells without antigen (blank), secondary antibody only (AF488 only) and BSA protein. Error bars depict SEM, *n* = 3. **(C)**: Representative images of undifferentiated MEL1 human ES cell cultured on a feeder layer of mouse embryonic fibroblasts in hPSC medium for 4‐7 days, showing surface immunostaining using purified anti‐hGPR64, anti‐hCDCP1, anti‐hF11R, anti‐hDSG2, anti‐hCDH3, anti‐hNLGN4X, anti‐hPCDH1 (green, AF488), merged with 4′,6‐diamidino‐2phenylindole (DAPI) counterstained nuclei (blue) and colocalizing with OCT4‐positive cells (red), compared with CD9, TRA‐1‐60 (AF488/DAPI), and isotype controls for the fluorochromes AF488 and AF647. All images shown are for fixed cell mAb staining, except for anti‐hGPR64‐AF488 that was live cell stained prior to fixation for OCT4 detection. Scale bars = 100 µm. Abbreviations: DAPI, 4′,6‐diamidino‐2phenylindole; FACS, fluorescence activated cell sorting; hPSCs, human pluripotent stem cells; mAbs, monoclonal antibodies.

To generate tools for detecting cell‐surface proteins on viable hPSCs that correlate with the presence of the pluripotency‐associated transcription factor OCT4 50, we selected candidate genes that we identified from our FACS‐based GCTM‐2/CD9 immunotranscriptional profiling of hPSCs 25. The workflow to obtain mAbs to these targets is outlined in Figure 1A. Briefly, we analyzed the protein domain structures for candidate markers and generated antigens via peptide synthesis or by protein expression in modified HEK293 cells for ∼30 cell‐surface proteins for which antibodies were either not commercially available at the time, or were available but did not detect epitopes on live hPSCs by flow cytometric analyses (data not shown). Following immunization with antigens and the generation of hybridomas, culture supernatants were screened by robotic solid‐state antigen array analyses for detection of the corresponding immunogen and then via high‐throughput flow cytometry to confirm capability for detecting live hPSCs (Supporting Information Fig. S1A). Of the 200‐300 hybridomas typically screened for each candidate protein, we observed that fewer than 10% subsequently detected cell‐surface protein on live hPSCs. Clonally expanded mAbs purified from hybridoma cultures (Supporting Information Fig. S1B) were further characterized in this study.

### New mAbs Detect Defined Cell Surface Proteins on HPSCs

**Figure 2:**
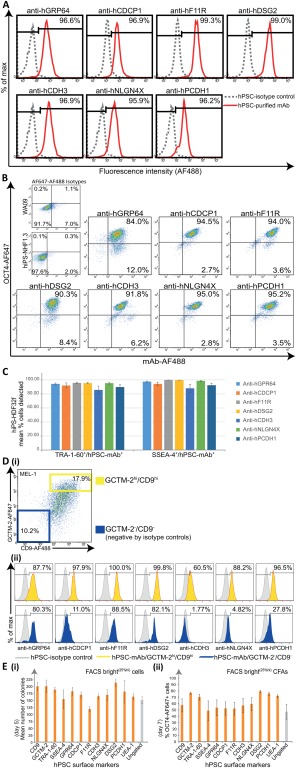
New monoclonal antibodies (mAbs) enable live cell detection and culture of self‐renewing human pluripotent stem cells (hPSCs). For undifferentiated hPSC cultures immunolabeled with new purified mAbs anti‐hGPR64, anti‐hCDCP1, anti‐hF11R, anti‐hDSG2, anti‐hCDH3, anti‐hNLGN4X, anti‐hPCDH1 **(A)** Representative flow cytometric histogram plots showing live cell fluorescence detection (AF488) of protein epitopes corresponding to all mAbs for a high percentage (%) of total MEL1 cells analyzed (hiPS‐NHF1.3 cells for anti‐hGPR64), (red histogram), against isotype controls (gray histogram). **(B)**: Representative flow cytometric dot plots showing high coexpression of mAb detected hPSC‐surface proteins (AF488) and OCT4 (AF647) against isotype controls following sequential live and fixed cell immunolabeling of hiPS‐NHF1.3 cells (anti‐hGPR64) and WA09 cells (all other mAbs). **(C)**: Multicolor immunostaining and flow cytometric replicate analyses shown graphically for the live cell codetection (mean %) of human iPS cells‐HDF32f cells by each mAb with the TRA‐1‐60 or SSEA‐4 pluripotency‐associated antibodies, compared with isotype controls (not shown). Bars represent the mean percentage of cells staining positively for each mAb within the TRA‐1‐60 or SSEA‐4 positive cell population (*n* = 3, mean +/− SEM). **(D)**: Triple color flow cytometric analyses demonstrates varying coexpression profiles of each mAb with the GCTM‐2/CD9 gradient in hPSC cultures; **(i)** Representative flow cytometric analysis for GCTM‐2 (AF647) and CD9 (AF488) coexpression gradient in live MEL1 cell cultures. Population gates are set against isotype controls for negative (GCTM‐2‐AF647^‐^/CD9‐AF488^‐^ blue gate) and high (GCTM‐2‐AF647^hi^/CD9‐AF488^hi^ yellow gate) cells; **(ii)** Representative flow cytometric histogram plots showing the percentage of mAb‐detected cells overlapping with the GCTM‐2/CD9 high (yellow) and negative (blue) populations against isotype controls (gray). **(E)**: Colony‐forming assays (CFAs) for the postfluorescence activated cell sorting (FACS) culture (5,000 cells/3.8cm^2^ well) of hiPS‐NHF1.3 cells gated for replating the top 25% (FACS bright25^%hi^, orange bars) and ungated (gray bar) cells detected by mAbs compared with CD9, GCTM‐2, TRA‐1‐60, SSEA‐4, and UEA‐1 lectin; **(i)** Mean colony counts per well after 5 days culture in hPSC conditions (*n* = 3). **(ii)** Percentage OCT4‐AF647 positive cells detected by flow cytometry for the preceding CFAs harvested at 7 days of post‐FACS culture for FACS bright25^%hi^ (orange bars) and ungated (gray bar) cells (*n* = 3). All Error bars depict SEM. Abbreviation: hPSCs, human pluripotent stem cells.

mAbs were raised against the following seven human recombinant proteins; CUB domain containing protein 1 (anti‐hCDCP1), platelet F11 receptor (anti‐hF11R), desmoglein 2 (anti‐hDSG2), cadherin 3 (anti‐hCDH3), neuroligin 4X‐linked (anti‐hNLGN4X) and protocadherin 1 (anti‐hPCDH1) and against a synthetic peptide sequence for G protein‐coupled receptor 64 isoform 4 (anti‐hGPR64), (Table 1 and Supporting Information Table S1). Antibodies were tested for their detection of live cells in undifferentiated hPSC cultures (see Fig. 2A). ELISA analyses (Fig. 1B) confirmed that the mAbs specifically detect each of the target cell‐surface proteins to which they were initially raised. ICC staining of fixed hPSCs (Fig. 1C) demonstrated that each of the newly generated mAbs displayed cell surface staining in undifferentiated cultures of MEL1 hESCs that were costained with OCT4, but not in the supporting mouse embryonic fibroblast (MEF) feeder cells (not shown). This staining is comparable to that observed for pluripotency‐associated markers TRA‐1‐60 and CD9 (Fig. 1C). For the anti‐hGPR64 mAb we observed the same strong cell surface staining as for the other mAbs for live hPSCs (Fig. 1C) but less consistently for fixed cell staining, suggesting that fixation alters the epitope recognition sequence for the peptide antigen to which this mAb was raised.

**Table 1:** Human cell surface proteins corresponding to new hPSC monoclonal antibodies

Gene	Alternate gene names	Common protein names
*GPR64*	EDDM6, HE6, TM7LN2	G protein‐coupled receptor 64 isoform 4
*CDCP1*	CD318, SIMA135, TRASK	CUB domain containing protein 1 isoform 1, membrane glycoprotein gp140
*F11R*	CD321, JAM, JAM1, JAMA, JCAM, KAT, PAM‐1	Junctional adhesion molecule A (JAM‐A), Platelet F11 receptor
*DSG2*	ARVC10, ARVD10, CDHF5, CMD1BB, HDGC	Desmoglein 2 (DSG2); cadherin family member 5
*CDH3*	CDHP, HJMD, PCAD	Cadherin 3 (CDH3), placental cadherin (P‐cadherin),
*NLGN4X*	ASPGX2, AUTSX2, HLNX, HNL4X, NLGN4	Neuroligin‐4 X‐linked (NLGN4X), neuroligin‐X
*PCDH1*	PC42, PCDH42	Protocadherin 1 isoform 1 (PCDH1), cadherin‐like protein 1.

**Figure 3:**
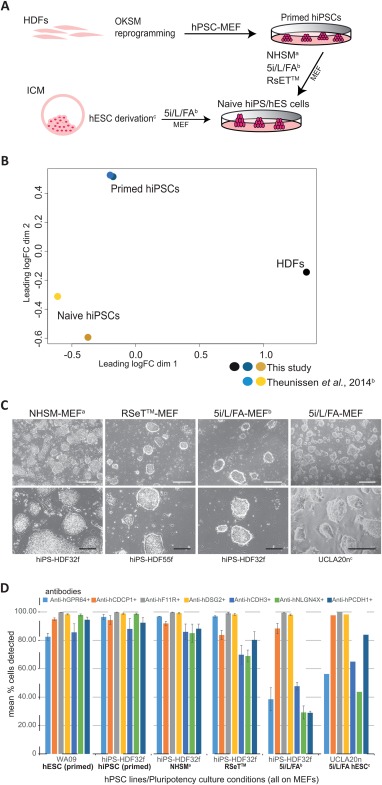
Cell surface antigens are detected on naive state human pluripotent cells. Transcriptional and protein analyses showing the expression of cell surface epitopes on human iPS cells (hiPSCs) and human ES cells (hESCs) cultured in conditions supporting a naive state of pluripotency. **(A)**: Schematic depicting the generation of naive human cell cultures both from lineage primed hiPSCs and blastocyst epiblast cells. Human dermal fibroblasts (HDFs) were reprogrammed to primed hiPSCs in standard human pluripotent stem cell (hPSC)‐MEF culture then coaxed to a naive pluripotent state in NHSM^a^, RSET, and 5i/L/FA^b^ defined media‐MEF supported culture conditions. Naive state hESCs were derived^c^ and maintained following the direct culture of preimplantation blastocyst in 5i/L/FA‐MEF conditions. **(B)**: PCA of RNA sequencing data for the primed and naive (5i/L/FA) state hiPSCs from this study and the microarray data extracted from the published report of Theunissen et al. (2014), 38 confirms differential clustering of two parental HDFs (black dot), lineage primed hiPSCs from this study (dark blue dot) and Theunissen et al. (2014), (light blue dot), and naive hiPSCs from this study (dark yellow dot) clustering with the naive hiPSCs from Theunissen et al. (2014) (gold dot). **(C)**: Representative images for naive hiPSC colonies originating from two parental HDF cell lines (HDF32f, HDF55f) following culture in NHSM^a^‐MEF, RSeT‐MEF, and 5i/L/FA^b^‐MEF conditions and preimplantation blastocyst‐derived hES naive cell colonies (UCLA20n^c^) generated directly in 5i/L/FA‐MEF culture conditions. HiPS and hESC naive cell cultures show a domed colony morphology by bright field phase contrast microscopy (BF). **(D)**: Flow cytometric replicate analyses shown graphically for the live cell detection of naive state hiPS‐HDF32f cells maintained on MEFs in NHSM^a^, 5i/L/FA^b^, and RSeT culture media, and naive UCLA20n hESCs in 5i/L/FA‐MEF culture, by the monoclonal antibodies anti‐hGPR64 (light blue bars), anti‐hCDCP1 (orange bars), anti‐hF11R (gray bars), anti‐hDSG2 (yellow bars), anti‐hCDH3 (mid blue bars), anti‐hNLGN4X (green bars), anti‐hPCDH1 (dark blue bars), compared with lineage primed WA09 cells (hESC primed) and parental hiPS‐HDF32f cells (hiPSC primed) cultured on MEFs in hPSC medium, against isotype controls (not shown), (*n* = 3, except UCLA20n in 5i/L/FA *n* = 2). Error bars depict SEM. Scale bars = 500 µm (white) and 200 µm (black) for BF images. ^a^Gafni O et al. Nature 2013;504:282‐286. ^b^Theunissen TW et al. Cell Stem Cell 2014;15:471‐487. ^c^Pastor WA et al. Cell Stem Cell 2016;18:323‐329. Abbreviations: hESCs, human ES cells; hiPSCs, human iPS cells.

To determine the broader utility of the new panel of purified mAbs we carried out a series of experiments using the following cell lines; MEL1 and WA09 (hESC lines) 1, 31, hiPS‐PDL‐D1C6 and hiPS‐NHF1.3 (hiPSC lines generated via lentiviral and episomal vector strategies, respectively, 32, 33) and the hiPS‐HDF cell lines generated in this study (see Materials and Methods). All mAbs recognized a high proportion of cells from all cell lines following live cell extracellular immunostaining and flow cytometry (Figs. 2A, 3D and Supporting Information Figs. S2A, S3B). Interestingly, a lesser average proportion of cells was detected by the anti‐hCDH3 mAb for two of the hiPSC cultures (70.25% ± 6.99% hiPS‐NHF1.3 and 69.86% ± 9.71% hiPS‐PDL‐D1C6 cells) compared with that detected for hiPS‐HDF32f cell cultures (88.08% ± 7.23%) and the hESC lines (87.90% ± 5.84% MEL1 and 90.66% ± 5.38% WA09), (Fig. 3D and Supporting Information Figs. S2A, S3B). This may be due to a higher level of heterogeneity, perhaps from differentiation, in the NHF1.3 and PDLD1C6 hiPS cell lines. Note, the seven mAbs also showed similar patterns of immunoreactivity as assessed by flow cytometry on hPSCs grown in Essential E8 Medium on a Geltrex Matrix (all from Life Technologies) 34, 35, (data not shown).

### New mAbs Show High Correlation with Immunodetection of OCT4 and Pluripotency‐Associated Antibodies in HPSC Lines

It is widely established that undifferentiated hPSC cultures will inherently be subject to a low level of spontaneous differentiation and accordingly vary in the percentage of cells showing OCT4 and cell surface marker immunoreactivity among cell lines and for each passage. We, therefore, determined the correlation of immunodetection of each mAb with OCT4, TRA‐1‐60, or SSEA‐4 using sequential immunostaining and flow cytometric analyses. Representative flow cytometric plots are shown for OCT4 double staining (Fig. 2B) and for TRA‐1‐60 and SSEA‐4 double staining with each mAb (Supporting Information Fig. S3). Replicate multicolour flow analyses (presented as bar graphs of mean percentages for each mAb of human OCT‐4 positive cells detected) confirmed a high correlation for detection of the OCT4 positive cells by each mAb on multiple hPSC lines (Supporting Information Fig. S2B, S2C), and similarly for hPSCs costained with each mAb and TRA‐1‐60 or SSEA‐4 (Fig. 2C). Collectively these results showed a very high level of concordance between staining for each of the seven new mAbs described herein and OCT‐4, TRA‐1‐60, or SSEA‐4 immunoreactivity. The results also demonstrated variation in the percentages of cells staining positively for all antibodies among individual cell populations, highlighting the necessity for comprehensive live cell characterization of hPSCs to avoid heterogeneous inputs to cell‐based applications, potentially leading to inefficient differentiation 5, 51.

We and others have shown in previous work that a GCTM‐2^neg^/CD‐9^neg^ subpopulation in hPSC cultures is associated with the very earliest spontaneous hPSC differentiation and this population does not yield teratomas following in vivo transplant 23, 24, 25, 41, 52. The variation for each mAb's immunoreactivity within this double‐negative population is predicted to reflect how rapidly the corresponding protein epitopes are downregulated during early stage hPSC differentiation. To investigate the association of our new antibodies with early lineage commitment of hPSCs, we determined the correlation between cell populations detected by each mAb and the GCTM‐2/CD9 profile of hPSCs (Fig. 2D, i‐ii). These analyses (Supporting Information Fig. S4) revealed that the mAb‐positive cell populations overlapped with ≥93.3% ± 5.59% of the GCTM‐2^hi^/CD‐9^hi^ (undifferentiated) population in MEL1 cultures, except for the CDH3 mAb, which detected an average of 72.50% ± 11.71% of cells in the GCTM‐2^hi^/CD‐9^hi^ subfraction. Of interest were the much greater differences seen among the mAbs in detection of cells in the GCTM‐2^neg^/CD‐9^neg^ gated (early differentiation) population, with anti‐hCDH3 detecting the average lowest number of cells (4.03% ± 2.53%) and anti‐hF11R detecting the average highest number of cells (87.70% ± 5.26%). Each mAb will, therefore, have specific utility as a pluripotency marker in studies seeking to resolve heterogeneity in undifferentiated hPSC cultures 26, 27, 28 as well as those interrogating in vitro recapitulation 53 or reprogramming 54 of early developmental events.

### New mAbs Enable Cell Sorting to Produce HPSC Colonies

We next sought to assess the utility of the new panel of mAbs for applications requiring viable hPSC detection and downstream culture. Colony‐forming assays (CFAs) were performed, to determine retention or loss of self‐renewal ability for hPSC populations detected by live cell FACS for each mAb, by replating cells gated for the brightest positive quartile population (mAb^25^ ^%hi^) into hPSC culture conditions. Visual morphological assessment at 5 days postplating confirmed the robust establishment of self‐renewing colonies containing cells exhibiting typical hPSC appearance of rounded, compact, high nuclear‐cytoplasmic ratio (not shown) from all mAb^25^ ^%hi^‐fractionated cells (Fig. 2E, i). Colony numbers ranged from an average minimum 119 ± 15 colonies/well for F11R^25%hi^ cells to an average maximum 214 ± 23 colonies for DSG2^25%hi^, compared with the widely used hPSC marker TRA‐1‐60 (189 ± 16 colonies), the lectin UEA‐1 (164 ± 19 colonies), and unfractionated hPSCs (151 ± 22 colonies) for the same cultures. Flow cytometric OCT4 analysis of colony cultures at day 7 (Fig. 2E, ii) determined that colonies formed from post‐FACS replating of mAb‐bright cells contained self‐renewing OCT4‐positive cells in percentages greater than that for unfractionated hPSCs, and comparable to that for UEA‐1, TRA‐1‐60, and SSEA‐4.

### Naive State Human Pluripotent Cells Express Antigens Detected by the New mAbs

Recent reports indicate that human PSCs can exist in two distinct pluripotent states. One state, termed “primed” is thought to be similar to that of murine postimplantation epiblast cells 20. The other state, termed “naive” or “ground state,” appears to be analogous to ICM‐derived murine cells 21, 55. Multiple groups have recently reported the generation of human PSCs from either blastocysts or by somatic cell reprogramming that bear a naive state phenotype 29, 39. Further, culture conditions supporting the demonstration of naive hPSCs from single human ICM cells have recently been reported 56. In this study, we asked whether the epitopes for any of our new mAbs were also detectable in naive states of pluripotency. We generated hiPSCs by reprogramming human dermal fibroblast (HDF) cells from two donors (HDF32f, HDF55f) using standard hPSC culture conditions that produce lineage primed hiPS cells, then cultured these in previously described NHSM defined culture conditions 37 5i/L/FA 38 and the recently available RSET to convert cells to a distinct naive cellular state. Immunoreactivity for each mAb was analyzed on these naïve hiPS cultures and also compared to mAb immunoreactivity on a recently published naive hESC line (UCLA20n), derived and cultured in the same 5i/L/FA conditions 39. Figure 3A summarizes the naive hPSC lines used in this study. RNA sequencing and metadata analyses confirmed distinct expression profiles for parental HDFs, primed and naive hiPSCs (5i/L/FA) that also clustered with published primed and naive cell data 38 (Fig. 3B, Supporting Information Fig. S5). Analysis of selected genes in our cultures demonstrated a high concordance with those reported for primed and naive state hiPSCs 38 (Supporting Information Fig. S5). Morphologically, hiPSC and hESC cultures maintained in each of the NHSM, 5i/L/FA and RSeT conditions, all displayed the domed morphology typically described for naive‐like pluripotent cells (Fig. 3C). The new panel of mAbs detecting cell surface epitopes in our primed hESC and parental HDF‐iPS cultures also demonstrated heterogeneity in detection of corresponding epitopes in the undifferentiated naive hiPSC cultures from the different culture methodologies (Fig. 3D). Interestingly the percentage of naive state hiPSC or hESC displaying positive immunoreactivity to four mAbs (hGPR64, hCDH3, hNLGN4X, & hPCDH1) cultured in 5i/L/FA conditions was consistently lower (but not absent) than for primed state hESC (less so for hPCDH1) or hiPSC. These results also indicate phenotypic differences between the hPSCs cultured in 5i/L/FA conditions and NHSM or RSeT cultured naive hPSCs. The NHSM cells showed very similar phenotypic immunoreactivity to the mAbs when compared to primed hPSCs whereas RSET cells show lower percentages of immunoreactivity to three mAbs (hCDH3, hNLGN4X, & hPCDH1) than primed cells (although not as low as 5i/L/FA cultured cells). It is interesting to speculate that loss of immunoreactivity to the four mAbs (hGPR64, hCDH3, hNLGN4X, & hPCDH1) indicates progression from a primed cell state to a more naive state but further experimentation is needed to determine whether loss of the epitopes detected by the mAbs is intrinsic to naive cells or not. The results also clearly indicate that three mAbs (hCDCP1, hF11R, & hDSG2) consistently stain greater than 80% of the cells cultured in naive or primed conditions. Collectively, these results demonstrate heterogeneity within naive cells produced using different culture methodologies. Pastor et al. (2016) 39 report that a subpopulation of SSEA‐4 negative cells more closely resemble the human preimplantation epiblast than do SSEA‐4 positive cells 57. We, therefore, further examined the immunoreactivity of each or our new mAbs with SSEA‐4 negative populations from 5i/L/FA cultured naive hiPSC (Supporting Information Fig. S6). Note the UCLA20n line is essentially SSEA‐4 negative (data not shown), as previously reported 39, and can, therefore, also be analyzed for comparison (Fig. 3D). The comparison of the SSEA‐4 negative population with the SSEA4 positive population of 5i/L/FA cultured naive hiPSCs and the UCLA20n cells indicates similar patterns of immunoreactivity implying that our new mAbs are not able to distinguish between these two populations (Supporting Information Fig. S6). Nevertheless, the above set of results examining human naive cell populations indicate that the new mAbs described herein will likely be useful tools for subfractionating these cell populations for further study of heterogeneity within naive states of human pluripotency and for studying differences between naive cells produced using distinct culture methodologies.

### Ability of New mAbs to Detect HPSCs During EB Differentiation

**Figure 4:**
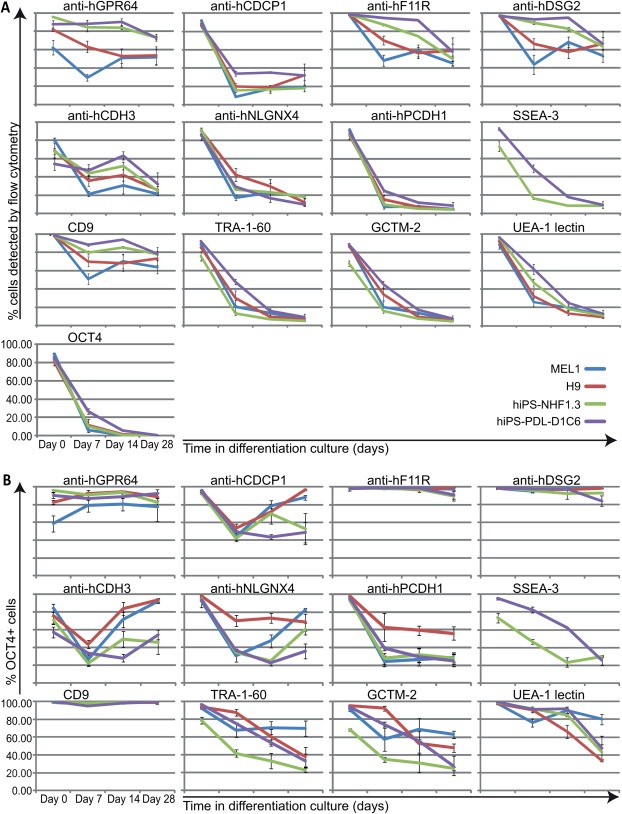
New monoclonal antibodies (mAbs) determine downregulation of cell surface epitopes during human pluripotent stem cells (hPSC) differentiation. **(A)**: Flow cytometric replicate analyses shown graphically for the detection of live cells by immunostaining with purified mAbs in differentiating embryoid body (EB) cultures for hPSC lines MEL1 (blue plotted lines), WA09 (red plotted lines), hiPS‐NHF1.3 (green plotted lines), hiPS‐PDL‐D1C6 (purple plotted lines) on the day of harvesting routine undifferentiated cultures (day 0), and following induction of differentiation in EB suspension cultures (days 7, 14, 28). Comparative analyses show detection of live cells for the same cultures and time points using CD9, TRA‐1‐60, GCTM‐2, and biotinylated UEA‐1 lectin, and fixed cell detection for OCT4 (*n* = 3). **(B)**: For the analyses shown in (A), data displayed graphically for % of total residual OCT4‐positive cells detected by each mAb and pluripotency associated marker in differentiating cultures for each cell line at each time point (*n* = 3). All error bars depict SEM.

Next, we assessed the immunoreactivity to cell‐surface proteins detected by each mAb in hPSC cultures undergoing spontaneous in vitro EB‐based differentiation, determined at 0, 7, 14, and 28 days. Flow cytometric analyses for each mAb in differentiation cultures for two hESC and two hiPSC lines were compared with CD9, TRA‐1‐60, UEA‐1, and OCT4 (Fig. 4A) and demonstrated a change in the percentage of cells displaying immunoreactivity of all hPSC markers but with differing kinetics seen over the time course. Concordant with the ability to detect GCTM‐2^neg^/CD9^neg^ cells in differentiating hPSC cultures (Fig. 2D, i‐ii, Supporting Information Fig. S4), the F11R, DSG2, and GRP64 mAbs displayed an expression profile in differentiating cultures that is downregulated but less rapidly than for the CDCP1, CDH3, NLGNX4, and PCDH1 mAbs, which show profiles comparative to those for OCT4, TRA‐1‐60, SSEA‐3, GCTM‐2, and UEA‐1 markers. CD9, after an initial downregulation at 7 days, retained the highest cell detection of all markers analyzed in this time course study, consistent with its known expression in epithelial cells 58. We further interrogated the flow cytometric analyses for EB cultures to determine the percentage of cells codetected by each marker of the OCT4‐labeled population at each time point (Fig. 4B, Supporting Information Table S3). Note this population becomes increasingly rarer over time. Following a rapid decline in OCT4 expression after 7 days differentiation for all cell lines, those mAbs displaying decreased but lingering detection of cell‐surface proteins after 28 days of differentiation (F11R, DSG2, GPR64, Fig. 4A) were also detecting the high percentages of residual OCT4‐positive cells. This underscores the potential ability of these three mAbs to remove unwanted residual OCT4‐positive cells from mixed differentiated cell populations.

### Broader Utility of New mAbs

The cell‐surface proteins in this study are all known for biological profiles that are of interest because of their association with development and disease states. Some of these markers are predicted to be misregulated in the onset of some types of cancers 59, 60. As one example, the F11R protein (also known as JAM‐A) is known to be critical for tight junction functioning in the developing blastocyst 61, and is implicated in regulating the migration of endothelial cells in the progression of various human malignancies including breast, gastric, and lung tumours 62, 63, 64. In addition, these proteins have interesting tissue‐ and cancer‐specific expression profiles reported in the *The Human Protein Atlas* 65 (http://www.proteinatlas.org). These specific expression patterns indicate that the mAbs developed in this study will be useful tools for examination of a range of human developmental processes and diseases.

We investigated the utility of the new pluripotency‐associated mAbs to interrogate detection or absence of epitopes expressed on MSCs derived from human bone marrow, and on epithelial and stromal cell populations isolated from human breast and epithelial cell populations from colon tissues. For hBM‐MSC cultures confirmed for a CD90^+^ phenotype, we found the anti‐hCDCP1 mAb detected the antigen on a subset of cells (50.2% ± SD 8.1%, data not shown). The CDCP1 antigen has been previously reported to be expressed in normal epithelial cells, overexpressed in proliferating epithelial tumors such as colon, breast, lung, renal cancers 66, 67, 68, and is suggested as a potential therapeutic target for pancreatic tumor cell migration and metastasis 69. More recently, CDCP1 is reported to be expressed on a functionally distinct CD146^neg^ subset of marrow fibroblasts that may play a role in regulating hematopoietic cytokine expression 70.

**Figure 5:**
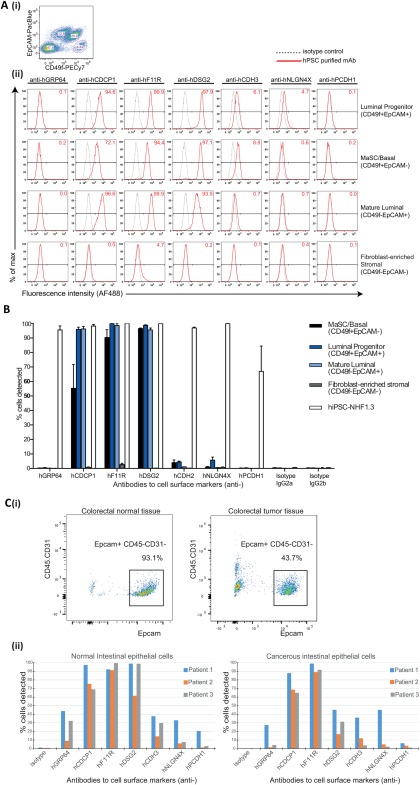
Expression of pluripotency‐associated antigens in human breast tissue and on normal and cancerous intestinal epithelial cells. **(A)**: Human CD31^−^CD45^−^CD235a^−^ cell populations isolated from mammary tissue were **(i)** fluorescence activated cell sorting (FACS)‐delineated using EpCAM and CD49f to resolve each of the four breast epithelial cell subsets:‐ luminal progenitor (CD49f^+^ EpCAM^+^), MaSC and basal (CD49f^+^ EpCAM^−^), mature luminal (CD49f^−^EpCAM^+^), and fibroblast‐enriched stromal (CD49f^−^EpCAM^−^); **(ii)** Representative flow histogram plots show the presence or absence of antigens detected by the purified monoclonal antibodies (mAbs) anti‐hGRP64, anti‐hCDCP1, anti‐hF11R, anti‐hDSG2, anti‐hCDH2, anti‐hNLGN4X, and anti‐hPCDH1 in these cell subsets, compared with isotype controls. **(B)**: Bar graphs show the detection of pluripotency‐associated antigens for mammary cell subsets from four donor specimens as mean +/− SEM across two technical replicates compared with isotype controls and NHF1‐3 hiPS positive control cells. **(C)**: (i) Human intestinal epithelial cells were isolated from normal and cancerous colorectal tissues by FACS selection for EpCAM^+^ CD31^−^CD45^−^ cells. (ii) Bar graphs show the variation in detection of pluripotency‐associated antigens by the panel of mAbs (A‐B) on normal and cancerous intestinal epithelial cells from three patient donor samples compared with isotype controls. Abbreviation: hiPSCs, human iPS cells.

Our study identified three antigens (CDCP1, F11R, and DSG2) that were highly expressed by human mammary epithelial cells following FACS into four unique subsets:‐ luminal progenitor (CD49f^+^ EpCAM^+^), mature luminal (CD49f^−^EpCAM^+^), MaSC and basal (CD49f^+^ EpCAM^−^) and fibroblast‐enriched stromal (CD49f^−^EpCAM^−^), (Fig. 5A, i‐ii). The luminal progenitor subset highly expressed F11R (99.9% ± 0.1%), DSG2 (98.7% ± 0.7%), and CDCP1 (95.9% ± 3.0%) and expressed low levels of NLGN4X (5.7% ± 4.1%) and CDH3 (4.3% ± 1.3%). The mature luminal subset highly expressed F11R (98.6% ± 2.7%), DSG2 (95.8% ± 2.2%) and CDCP1 (96.2% ± 3.6%), and low levels of CDH3 (1.0% ± 0.3%). The MaSC/basal subset highly expressed F11R (90.4% ± 10.7%) and DSG2 (96.5% ± 0.8%) and expressed CDCP1 to some extent (55.3% ± 32.7%) as well as weak expression of CDH3 (4.0% ± 3.7%). The fibroblast‐enriched stromal population expressed very low levels (2.7% ± 1.8%) of F11R on cells and was devoid of all other antigens. Our mAbs did not detect PCDH1 nor GRP64 expression on any of the human breast epithelial or stromal subsets (Fig. 5A, ii, 5B). Collectively these results show the ability of the pluripotency‐associated antibodies to specifically discern antigen expression on human breast epithelial and stromal subpopulations and raise the possibility that these antigens may have potential as biomarkers for breast cancer. Extending from the above reports exemplifying CDCP1 overexpression on cancerous epithelial cells from multiple tissues 66, 67, 68, 69, a recent study indicates CDCP1 as a modulator of HER2 signaling, an interaction that results in increased tumor formation and cell migration as well as c‐SRC mediated resistance to trastuzumab in HER2‐positive breast cancer patients 71. F11R is reported to be expressed in the luminal and basal cells of normal human breast tissue and is overexpressed in breast tumours, in which it correlates with a poor prognosis 72, 73, 74. The downregulation of F11R expression in murine and human mammary tumor models has been shown to reduce tumor proliferation by increasing cell susceptibility to apoptosis 73, 74. DSG2 is a key glycoprotein found in the desmosomes of epithelial junctions and is localized to both the luminal and myoepithelium of normal breast tissue 75. As most solid cancers are of epithelial origin, DSG2 is consistently upregulated in most cancers analyzed 65. The maintenance of intercellular junctions in both normal epithelial and tumor tissues acts to prevent host immune responses and creates a barrier that prevents the effective dissemination of cancer therapeutics 76.

Our analyses of an enriched EpCAM^+^ (CD45^−^CD31^−^) intestinal epithelial (IE) cell population 43 (Fig. 5C, i) isolated from the normal colon and colorectal cancer tissues of three patients demonstrated firstly, the overall reduction in IE cell retrieval from cancerous tissues, and second, the detection to varying degrees of all epitopes on normal IE cells corresponding to the panel of pluripotency mAbs, excepting the very low expression of PCDH1 for two patient IE populations (Fig. 5C, ii). While detected antigen expression is variable between donor patient tissues (Fig. 5C, ii), the complete maintenance of upregulated CDCP1 and F11R expression in both normal and cancerous IE populations, is consistent with the aggressive proliferation of tumor cells in colorectal cancer 66, 67, 68, and the maintenance of tight junctions in both normal and dedifferentiated epithelial tumor cells 76. Interestingly, downregulation of DSG2 expression was demonstrated for the cancerous IE cells from all patients compared with their corresponding normal tissue IE populations. Markers detected in cell subsets of the normal IE populations of all patients (GRP64, CDH3, NLGN4X, and PCDH1) displayed a trend for downregulation in corresponding tumor IE cell samples, except for one patient's samples displaying some upregulation of NLGN4X (Fig. 5C, ii). NLGN4X is an adhesion molecule involved in neuronal cell adhesion and synaptic formation and function 77, with various mutations of the encoding gene being implicated in autism spectrum disorders 78. Evidence for NLGN4X expression in normal colorectal tissue is reported, but its overexpression in tumors is to date associated principally with gliomas, ovarian, endometrial, breast, and uroepithelial cancers 65. Collectively, these studies indicate the utility of antibody‐based in vitro interrogation of both normal developmental events and the exploration for biomarkers applicable to the stratification of individual patients in treating a wide range of cancers.

While published genomic‐ and proteomic‐based studies have previously identified some of these candidate extracellular hPSC markers 13, 59, to date the existing antibodies for these proteins, many of which are polyclonal, have not been demonstrated to effectively detect epitopes on live hPSCs. Since the mAbs reported in this study have been raised to known protein antigens and all were specifically screened for detection on live hPSCs, these mAbs provide a greatly expanded resource for a variety of applications, including purification of subpopulations, removal of unwanted cells from differentiating hPSC derivatives, and detailed study of the nature of pluripotency.

## Conclusion

In conclusion, we report the generation of a panel of new mAbs that can efficiently detect the presence of cell‐surface epitopes on viable human embryonic and induced pluripotent stem cells. Moreover, we demonstrate that our new panel of antibodies detect the expression of these proteins on subsets of human cells when derived using naive conditions or reset in vitro to a naive pluripotent state 29, 39, 56. We anticipate that the novel antibodies generated and validated in this study will be valuable tools for studying human pluripotency, cellular reprogramming and differentiation, and for development of strategies enabling stringent quality control of live hPSC‐derived cell populations destined for clinical use.

## Author Contributions

C.M.O'B.: conception and design, collection and assembly of data, data analysis and interpretation, manuscript writing; H.S.C.: collection and assembly of data, data analysis and interpretation, assistance with manuscript preparation; Q.Z., S.B., J.W.L., J.K., B.C., T.C., X.L., F.J.R., C.M.N., D.C., T.J.: collection and/or assembly of data, data analysis and interpretation; T.E.A., T.P., J.D.B., W.J.M., Y‐C.W., K.O, P.J.M., G.J.L.: provision of study material; A.T.C., H.E.A., J.E.V., J.M.P.: provision of study material, data analysis and interpretation; J.F.L. financial support, data analysis and interpretation, manuscript writing; A.L.L.: conception and design, financial support, data analysis and interpretation, manuscript writing, final approval of manuscript.

## Disclosure of Potential Conflicts of Interest

The authors indicate no potential conflicts of interest.

## Supporting information

Supporting Information Figure 1.

Supporting Information Figure 2.

Supporting Information Figure 3.

Supporting Information Figure 4.

Supporting Information Figure 5.

Supporting Information Figure 6.

Supporting Information Figure 7.

**Supplemental Table S1**. Protein sequence information used to generate antigens corresponding to predicted hPSC surface epitopes.

**Supplemental Table S2.** Antibodies & conjugated fluorochrome reagents used in this study

Supporting Information Table 3.
