# Supplementary material for: New Monoclonal Antibodies to Defined Cell Surface Proteins on Human Pluripotent Stem Cells
Source: Stem Cells. 2017 Jan 19;35(3):626–40. doi: 10.1002/stem.2558 (PMC5412944; doi:10.1002/stem.2558)
Supplement: Supplementary file 8 — Supplemental Table S1. Protein sequence information used to generate antigens corresponding to predicted hPSC surface epitopes. [file STEM-35-626-s008.docx]

| GENE | Genebank Accession  number | Gene synonyms | Common protein names | Extracellular domain for antigen generation | Amino acid sequence |
| --- | --- | --- | --- | --- | --- |
| *GPR64* | NM_005756.3 | *EDDM6*  *HE6*  *TM7LN2* | G protein-coupled receptor 64 (GPR64) isoform 4 | ECD 3  ~778-789 | CWINNNAVFYIT (peptide synthesis) |
| *CDCP1* | NM_022842.4 | *CD318*  *SIMA135*  *TRASK* | CUB domain containing protein 1 (CDCP1); membrane glycoprotein gp140 | ECD 1  30~667 | FEIALPRESNITVLIKLGTPTLLAKPCYIVISKRHITMLSIKSGERIVFTFSCQSPENHFVIEIQKNIDCMSGPCPFGEVQLQPSTSLLPTLNRTFIWDVKAHKSIGLELQFSIPRLRQIGPGESCPDGVTHSISGRIDATVVRIGTFCSNGTVSRIKMQEGVKMALHLPWFHPRNVSGFSIANRSSIKRLCIIESVFEGEGSATLMSANYPEGFPEDELMTWQFVVPAHLRASVSFLNFNLSNCERKEERVEYYIPGSTTNPEVFKLEDKQPGNMAGNFNLSLQGCDQDAQSPGILRLQFQVLVQHPQNESNKIYVVDLSNERAMSLTIEPRPVKQSRKFVPGCFVCLESRTCSSNLTLTSGSKHKISFLCDDLTRLWMNVEKTISCTDHRYCQRKSYSLQVPSDILHLPVELHDFSWKLLVPKDRLSLVLVPAQKLQQHTHEKPCNTSFSYLVASAIPSQDLYFGSFCPGGSIKQIQVKQNISVTLRTFAPSFRQEASRQGLTVSFIPYFKEEGVFTVTPDTKSKVYLRTPNWDRGLPSLTSVSWNISVPRDQVACLTFFKERSGVVCQTGRAFMIIQEQRTRAEEIFSLDEDVLPKPSFHHHSFWVNISNCSPTSGKQLDLLFSVTLTPRTVDLT |
| *F11R* | NM_016946.4 | *CD321*  *JAM*  *JAM1*  *JAMA*  *JCAM*  *KAT*  *PAM-1* | Platelet F11 receptor (F11R); Junctional adhesion molecule A (JAM-A) | ECD 1  28~238 | SVTVHSSEPEVRIPENNPVKLSCAYSGFSSPRVEWKFDQGDTTRLVCYNNKITASYEDRVTFLPTGITFKSVTREDTGTYTCMVSEEGGNSYGEVKVKLIVLVPPSKPTVNIPSSATIGNRAVLTCSEQDGSPPSEYTWFKDGIVMPTNPKSTRAFSNSSYVLNPTTGELVFDPLSASDTGEYSCEARNGYGTPMTSNAVRMEAVERNVGV |
| GENE | Genebank Accession | Gene synonyms | Common protein names | Extracellular domain for antigen generation | Amino acid sequence |
| *DSG2* | NM_001943.3 | *ARVC10*  *ARVD10*  *CDHF5*  *CMD1BB*  *HDGC* | Desmoglein 2 (DSG2); cadherin family member 5 | ECD 1  50~609 | AWITAPVALREGEDLSKKNPIAKIHSDLAEERGLKITYKYTGKGITEPPFGIFVFNKDTGELNVTSILDREETPFFLLTGYALDARGNNVEKPLELRIKVLDINDNEPVFTQDVFVGSVEELSAAHTLVMKINATDADEPNTLNSKISYRIVSLEPAYPPVFYLNKDTGEIYTTSVTLDREEHSSYTLTVEARDGNGEVTDKPVKQAQVQIRILDVNDNIPVVENKVLEGMVEENQVNVEVTRIKVFDADEIGSDNWLANFTFASGNEGGYFHIETDAQTNEGIVTLIKEVDYEEMKNLDFSVIVANKAAFHKSIRSKYKPTPIPIKVKVKNVKEGIHFKSSVISIYVSESMDRSSKGQIIGNFQAFDEDTGLPAHARYVKLEDRDNWISVDSVTSEIKLAKLPDFESRYVQNGTYTVKIVAISEDYPRKTITGTVLINVEDINDNCPTLIEPVQTICHDAEYVNVTAEDLDGHPNSGPFSFSVIDKPPGMAEKWKIARQESTSVLLQQSEKKLGRSEIQFLISDNQGFSCPEKQVLTLTVCECLHGSGCREAQHDSYVG |
| *CDH3* | NM_001793.4 | *CDHP*  *HJMD*  *PCAD* | Cadherin 3 (CDH3), placentalcadherin (P-cadherin) | ECD 1  108~654 | DWVVAPISVPENGKGPFPQRLNQLKSNKDRDTKIFYSITGPGADSPPEGVFAVEKETGWLLLNKPLDREEIAKYELFGHAVSENGASVEDPMNISIIVTDQNDHKPKFTQDTFRGSVLEGVLPGTSVMQVTATDEDDAIYTYNGVVAYSIHSQEPKDPHDLMFTIHRSTGTISVISSGLDREKVPEYTLTIQATDMDGDGSTTTAVAVVEILDANDNAPMFDPQKYEAHVPENAVGHEVQRLTVTDLDAPNSPAWRATYLIMGGDDGDHFTITTHPESNQGILTTRKGLDFEAKNQHTLYVEVTNEAPFVLKLPTSTATIVVHVEDVNEAPVFVPPSKVVEVQEGIPTGEPVCVYTAEDPDKENQKISYRILRDPAGWLAMDPDSGQVTAVGTLDREDEQFVRNNIYEVMVLAMDNGSPPTTGTGTLLLTLIDVNDHGPVPEPRQITICNQSPVRQVLNITDKDLSPHTSPFQAQLTDDSDIYWTAEVNEEGDTVVLSLKKFLKQDTYDVHLSLSDHGNKEQLTVIRATVCDCHGHVETCPGPWKGG |
| GENE | Genebank Accession | Gene synonyms | Common protein names | Extracellular domain for antigen generation | Amino acid sequence |
| *NLGN4X* | NM_020742.3 | *ASPGX2*  *AUTSX2*  *HLNX*  *HNL4X*  *NLGN4* | Neuroligin 4 X-linked (NLGN4X); neuroligin-X | ECD 1  42~676 | QAQYPVVNTNYGKIRGLRTPLPNEILGPVEQYLGVPYASPPTGERRFQPPEPPSSWTGIRNTTQFAAVCPQHLDERSLLHDMLPIWFTANLDTLMTYVQDQNEDCLYLNIYVPTEDDIHDQNSKKPVMVYIHGGSYMEGTGNMIDGSILASYGNVIVITINYRLGILGFLSTGDQAAKGNYGLLDQIQALRWIEENVGAFGGDPKRVTIFGSGAGASCVSLLTLSHYSEGLFQKAIIQSGTALSSWAVNYQPAKYTRILADKVGCNMLDTTDMVECLRNKNYKELIQQTITPATYHIAFGPVIDGDVIPDDPQILMEQGEFLNYDIMLGVNQGEGLKFVDGIVDNEDGVTPNDFDFSVSNFVDNLYGYPEGKDTLRETIKFMYTDWADKENPETRRKTLVALFTDHQWVAPAVATADLHAQYGSPTYFYAFYHHCQSEMKPSWADSAHGDEVPYVFGIPMIGPTELFSCNFSKNDVMLSAVVMTYWTNFAKTGDPNQPVPQDTKFIHTKPNRFEEVAWSKYNPKDQLYLHIGLKPRVRDHYRATKVAFWLELVPHLHNLNEIFQYVSTTTKVPPPDMTSFPYGTRRSPAKIWPTTKRPAITPANNPKHSKDPHKTGPEDTTVLIETKRDYSTELS |
| *PCDH1* | NM_002587.4 | *PC42*  *PCDH42* | Protocadherin 1 (PCDH1); cadherin-like protein 1 | ECD 1  58~852 | TRVVYKVPEEQPPNTLIGSLAADYGFPDVGHLYKLEVGAPYLRVDGKTGDIFTTETSIDREGLRECQNQLPGDPCILEFEVSITDLVQNGSPRLLEGQIEVQDINDNTPNFASPVITLAIPENTNIGSLFPIPLASDRDAGPNGVASYELQAGPEAQELFGLQVAEDQEEKQPQLIVMGNLDRERWDSYDLTIKVQDGGSPPRASSALLRVTVLDTNDNAPKFERPSYEAELSENSPIGHSVIQVKANDSDQGANAEIEYTFHQAPEVVRRLLRLDRNTGLITVQGPVDREDLSTLRFSVLAKDRGTNPKSARAQVVVTVKDMNDNAPTIEIRGIGLVTHQDGMANISEDVAEETAVALVQVSDRDEGENAAVTCVVAGDVPFQLRQASETGSDSKKKYFLQTTTPLDYEKVKDYTIEIVAVDSGNPPLSSTNSLKVQVVDVNDNAPVFTQSVTEVAFPENNKPGEVIAEITASDADSGSNAELVYSLEPEPAAKGLFTISPETGEIQVKTSLDREQRESYELKVVAADRGSPSLQGTATVLVNVLDCNDNDPKFMLSGYNFSVMENMPALSPVGMVTVIDGDKGENAQVQLSVEQDNGDFVIQNGTGTILSSLSFDREQQSTYTFQLKAVDGGVPPRSAYVGVTINVLDENDNAPYITAPSNTSHKLLTPQTRLGETVSQVAAEDFDSGVNAELIYSIAGGNPYGLFQIGSHSGAITLEKEIERRHHGLHRLVVKVSDRGKPPRYGTALVHLYVNETLANRTLLETLLGHSLDTPLDIDIAGDPEYERSKQRGN |
