# Supplementary material for: New Monoclonal Antibodies to Defined Cell Surface Proteins on Human Pluripotent Stem Cells
Source: Stem Cells. 2017 Jan 19;35(3):626–40. doi: 10.1002/stem.2558 (PMC5412944; doi:10.1002/stem.2558)
Supplement: Supplementary file 9 — Supplemental Table S2. Antibodies & conjugated fluorochrome reagents used in this study [file STEM-35-626-s009.docx]

**Supplemental Table S2.** Antibodies & conjugated fluorochrome reagents used in this study

| Antibodies | Source | Host | Isotype | Working dilution; Concentration |
| --- | --- | --- | --- | --- |
| anti-human GPR64 | CSIRO  (CSTEM7) | Mouse | IgG2a, κ | 40 µg/ml |
| anti-human CDCP1 | CSIRO  (CSTEM26) | Mouse | IgG2a, κ | 20 µg/ml |
| anti-human F11R | CSIRO  (CSTEM27) | Mouse | IgG2a, κ | 4 µg/ml |
| anti-human DSG2 | CSIRO  (CSTEM28) | Mouse | IgG2b, κ | 3 µg/ml |
| anti-human CDH3 | CSIRO  (CSTEM29) | Mouse | IgG2a, κ | 20 µg/ml |
| anti-human NLGN4X | CSIRO  (CSTEM30) | Mouse | IgG2a, κ | 20 µg/ml |
| anti-human PCDH1 | CSIRO  (CSTEM31) | Mouse | IgG2b, κ | 20 µg/ml |
| CD9 | Purified monoclonal antibody. Kind gift from Prof. Martin Pera (University of Melbourne). | Mouse | IgG2a | 1:2000 |
| GCTM-2 | Monoclonal hybridoma supernatant. Kind gift from Prof. Martin Pera. | Mouse | IgM | 1:5 ~1:10^a^ |
| TRA-1-60 | Merck Millipore  Cat# MAB4360 | Mouse | IgM | 1:100 |
| SSEA-3 | Merck Millipore  Cat# MAB4303 | Rat | IgM | 1:100 |
| SSEA-4 | Merck Millipore  Cat# MAB4304 | Mouse | IgG3 | 1:100 |
| OCT4 | Merck Millipore  Cat# MAB4305 | Mouse | IgG1 | 1:250 |
| Purified Mouse IgG2a, κ isotype control | BD Biosciences  Cat# 554126 | Mouse | IgG2a | 1:100 |
| Purified Mouse IgG2b, κ isotype control | BD Biosciences  Cat# 559530 | Mouse | IgG2b | 1:100 |
| Purified Mouse IgG1, κ isotype control | BD Biosciences  Cat# 553447 | Mouse | IgG1 | 1:100 |
| Purified Mouse IgM, κ isotype control | BD Biosciences  Cat# 553472 | Mouse | IgM | 1:100 |
| Alexa Fluor® 488 conjugated Goat anti-mouse IgG (H+L) | Life Technologies  Cat# A11001 | Goat | Polyclonal | 1:500 |
| Alexa Fluor® 488 conjugated Goat anti-mouse IgM, µ | Life Technologies  Cat# A21042 | Goat | Polyclonal | 1:500 |
| Alexa Fluor® 647 conjugated Goat anti-mouse IgG1, γ_1_ | Life Technologies  Cat# A21240 | Goat | Polyclonal | 1:500 |
| Alexa Fluor® 488  Goat anti-rat IgM, µ | Life Technologies  Cat# A21212 | Goat | Polyclonal | 1:500 |
| R-Phycoerythrin (PE) conjugated Goat anti-mouse IgG2a, γ_2a_ | Life Technologies  Cat# P21139 | Goat | Polyclonal | 1:1,000 |
| Purified Rat IgG2a  isotype control | WEHI Monoclonal Antibody Facility | Rat | IgG2a | 1:40-2000 |
| Antibodies | Source | Host | Isotype | Working dilution; Concentration |
| PE conjugated Rat anti-mouse CD90.2 | BD Biosciences  Clone 30-H12 Cat# 553014 | Rat | IgG2b, κ | 1:100 |
| PE conjugated Mouse anti-human TRA-1-85 | R&D Systems  Clone TRA-1-85  Cat# FAB3195P | Mouse | IgG1 | 1:60 |
| APC conjugated Mouse anti-human CD90 | BD Pharmingen  Clone 5E10 Cat# 550402 | Mouse | IgG1, κ | 10 µg/ml |
| Biotinylated UEA-1 lectin | Provided by Loring Laboratory, TSRI. | n/a | n/a | 6.5 μg/ml |
| FITC Streptavidin | Life Technologies  Cat# SA1001 | n/a | n/a | 1:500 |
| Horizon^TM^ BUV395 conjugated TRA-1-60 | BD Biosciences  Clone TRA-1-60  Cat# 563878 | Mouse | IgM, k | 1:50 |
| PE-Vio770^TM^ conjugated SSEA-4 | Miltenyi Biotec  Clone REA101  Cat# 130-105-051 | Mouse | IgG1, k | 1:10 |
| PECy7 conjugated CD49f | eBioscience  Clone GoH3 Cat# 25-0495 | Rat | IgG2a, κ | 1:40-2000 |
| CD16/CD32 Fcg III/II receptor antibody | WEHI Monoclonal Antibody Facility  Clone 2.4G2-16 | Rat | IgG2b | 1:40-2000 |
| Pacific Blue^TM^ conjugated EpCAM | Cell Signaling Technology  Clone VU1D9 Cat# 9032 | Mouse | IgG1 | 1:40-2000 |
| Horizon^TM^ Brilliant Violet  BV421 conjugated EpCAM | BD Biosciences  Clone EBA-1 Cat# 563180 | Mouse | IgG1, λ | 1:200 |
| Brilliant Violet 421^TM^  conjugated EpCAM | BioLegend  Clone 9C4 Cat# 324220 | Mouse | IgG2b, κ | 1:100 |
| Purified Mouse anti-human CD235a | BD Pharmingen  Clone GA-R2 Cat# 555569 | Mouse | IgG2b, κ | 1:40-2000 |
| PE conjugated CD45 | BD Pharmingen  Clone H130 Cat# 555483 | Mouse | IgG1, κ | 1:40-2000 |
| Horizon^TM^ Brilliant Violet  BV510 conjugated CD45 | BD Biosciences  Clone HI30 Cat# 563204 | Mouse | IgG1, κ | 1:200 |
| PE conjugated CD31 | BD Pharmingen  Clone WM59 Cat# 555446 | Mouse | IgG1, κ | 1:40-2000 |
| Horizon^TM^ Brilliant Violet  BV510 conjugated CD31 | BD Biosciences  Clone WM59 Cat# 563454 | Mouse | IgG1, κ | 1:200 |

^a^ Zhou, Q., Chy, H. & Laslett, A.L. Preparation of defined human embryonic stem cell populations for transcriptional profiling. *Curr Protoc Stem Cell Biol* **Chapter 1**, Unit1B 7 (2010).
